# Exploration of Mobile Device Behavior for Mitigating Advanced Persistent Threats (APT): A Systematic Literature Review and Conceptual Framework

**DOI:** 10.3390/s22134662

**Published:** 2022-06-21

**Authors:** Thulfiqar Jabar, Manmeet Mahinderjit Singh

**Affiliations:** School of Computer Science, Universiti Sains Malaysia, Gelugor 11800, Penang, Malaysia; thulfiqar87@student.usm.my

**Keywords:** Situational Awareness (SA), Observe–Orient–Decide–Act (OODA), risk management, trust management, zero trust, threat modeling, fingerprint, security, privacy, Internet of Things (IoT)

## Abstract

During the last several years, the Internet of Things (IoT), fog computing, computer security, and cyber-attacks have all grown rapidly on a large scale. Examples of IoT include mobile devices such as tablets and smartphones. Attacks can take place that impact the confidentiality, integrity, and availability (CIA) of the information. One attack that occurs is Advanced Persistent Threat (APT). Attackers can manipulate a device’s behavior, applications, and services. Such manipulations lead to signification of a deviation from a known behavioral baseline for smartphones. In this study, the authors present a Systematic Literature Review (SLR) to provide a survey of the existing literature on APT defense mechanisms, find research gaps, and recommend future directions. The scope of this SLR covers a detailed analysis of most cybersecurity defense mechanisms and cutting-edge solutions. In this research, 112 papers published from 2011 until 2022 were analyzed. This review has explored different approaches used in cybersecurity and their effectiveness in defending against APT attacks. In a conclusion, we recommended a Situational Awareness (SA) model known as Observe–Orient–Decide–Act (OODA) to provide a comprehensive solution to monitor the device’s behavior for APT mitigation.

## 1. Introduction

The rapid expansion of the Internet of Things (IoT) and its ability to provide a broad variety of services make it the fastest-growing technology with a substantial impact on both business environments and social life [1]. Examples of IoT include mobile devices such as tablets and smartphones [2]. Smartphones have encroached on every aspect of modern life as they store personal and financial information, as well as information about companies and product marketing and development. However, the mobile nature of the smartphone, which means its physical location changes frequently [3], the diverse end-point devices with multiple Operating Systems (OS) and distributed heterogeneous networks [4,5], and the limited resources with restricted computing power, minimal storage capacity, and very specific energy resources [6,7], lead to a lack of security and privacy protection that can be embedded into the smartphone. As a result, it is easy for smartphones to suffer cyber and physical attacks [7].

One type of attack that occurs on a smartphone is known as Advanced Persistent Threats (APTs). This is a sophisticated and specific target attack with the aim of either data theft, disrupting the targeted system, or both [3]. In order to compromise the targeted system, APTs employs social engineering techniques to collect the required information about the target. APTs then employs either cyber techniques such as spear phishing and a watering hole or physical attacks to deliver the payload to the targeted system. Instead of directly executing a large number of activities, only a few essential activities are performed or conceal the payload [8]. A successful APT attack might persist for months or even years. A prime example is ZooPark, which is a cyberespionage toolkit that targeted android devices in 2015 and was active for three years until its discovery in 2018 [9]. The financial loss caused by APTs can be immense. According to Chainalysis [10], in 2021, at least seven cyber-attacks performed by North Korean cybercriminals targeted cryptocurrency platforms, aiming to steal digital assets with a value of $400 million. As reported in the Global Market Report for Advanced Persistent Threat Protection [11], the APT protection market was $7.2 billion in 2020 and is predicted to reach $21 billion in 2027, rising at a compound annual growth rate (CAGR) of 16.6% during the forecast period of 2020–2027.

An attacker can manipulate any device’s behavior, applications, and services depending on the goal, be it data theft or sabotage. This kind of manipulation results in a significant divergence from a known behavioral baseline, which may subsequently be utilized to identify a potential security risk. APTs can be tackled using different techniques such as Artificial Intelligence (AI), Machine Learning (ML), Deep Learning (DL), game theory, Situational Awareness (SA), risk management, trust management, and access control. In addition, device behavior-based detection techniques have been highlighted as one of the most promising approaches to address this issue [12]. It models the device’s behaviors and components to improve performance and detect potential attacks early based on previously recognized normal device behavior [12]. Device behavior detection solutions have focused on either soft computing techniques such as ML [13,14] or Intrusion Detection System (IDS) adaptation using anomaly detection [15,16,17]. Based on previous studies, most of the solutions have failed to tackle an APT issue using system behavior models because the existing detection solutions fail to map the behavior to the unique characteristics of APT attacks due to the following factors:

1. Some of the detection solutions lack APT detection for every stage of the attack life cycle. Work done by Mohammad and Belaton [13] focused on the credential dumping technique through monitoring CPU, RAM, Windows Registry, and file systems in order to detect APT. However, the authors only focused on one stage of the APT (credential access stage) and did not provide a comprehensive solution to detect APTs in all stages of the APT life cycle.
2. Some of the detection solutions are ineffective to detect an APT. Friedberg et al. [16] and Han et al. [17] proposed IDS to model the device behavior in order to detect APT using system events. However, these techniques might raise false positive alarms when normal system behavior changes.
3. Some of the detection solutions are inefficient in detecting APTs. Luh et al. [15] have proposed AIDIS, an Advanced Intrusion Detection and Interpretation System for APT detection and classification using Machine Learning techniques. However, this solution may not be capable of early detection of APTs.
4. Most APT detection solutions only focused on a group of users instead of individual user protection. Indeed, the risk associated with each device’s behavior varies according to the user’s behavior [18].
5. Most APT detection solutions fail to adopt any cyber security framework such as the National Institute of Standards and Technology (NIST) and the International Organization for Standardization (ISO) [19]. These detection solutions are not comprehensive to detect APT. NIST is an example of a cyber-security framework [20]. It categorizes the cybersecurity capabilities into five core functions (Identify, Protect, Detect, Respond, and Recovery) to organize and improve the cybersecurity models [20]. Based on NIST, most solutions fail to include the identify stage, which means the existing APT detection solutions are unable to quantify the risk related to the vulnerabilities of the attack. In addition, these APT solutions fail to include the protection stage as these solutions do not provide a function to prevent data leakage [21] or APT lateral movement [22].

In this study, the authors have analyzed the research published on APT defense mechanisms and identified the best possible defensive algorithms, frameworks, architectures, and models for various scenarios. The research objectives include the following: (1) Conduct a comprehensive systematic literature review (SLR) on mobile-based APT detection. It presents a general overview of the APT activities that targeted various environments, different defense algorithms, frameworks, architectures, and models that have tackled this issue, and a general overview of risk management models used to identify an APT. (2) Analyze the device’s behavior-based APT defense mechanisms. (3) Identify the key research challenges as well as future research directions. The main contributions of this SLR are to provide awareness to service owners, developers, and researchers of the issues surrounding the impact of APT attacks and APT defense mechanisms that focus on monitoring device behavior and its components.

Previous systematic reviews have made significant contributions to the cybersecurity field. The work of [23] has presented a general overview of the APTs and its communication mechanism that communicate the compromised host with the command and control (C&C) server where the persistent malware takes commands and harvested data is ex-filtrated. In addition, the authors have analyzed a few defense frameworks for APT detection and prevention from 2011 to 2017 and present the shortfalls of these frameworks. Furthermore, the authors have suggested to carry out an analysis and propose an APT defense framework for industrial control systems. This framework is a multilayer protection and detection system to protect the organization network to detect the APT only on one stage of the APT life cycle through the C&C stage. Our review has investigated and evaluated 112 journal papers from 2011 to 2022 to cover the great APT activity in the area via the formulation and answering of research questions. Different attacks that could be used by APT attacks to achieve their goal have been investigated. These attacks have been grouped based on the APT life cycle using a threat modeling approach such as MITRE ATT&CK. In addition, this review has investigated and evaluated different defense mechanisms utilized against APTs on devices and networks. Furthermore, this SLR has presented a general overview of the risk management approaches to identify APT. In the conclusion, the authors have filled the current research gaps by proposing a conceptual framework of mobile device behavior fingerprints for APT mitigation. This framework is used to protect and identify the suspicious activities in all APT life cycle stages through continuously monitoring the device behavior usage in order to overcome existing limitations in the literature.

This SLR comprises seven sections organized as follows: Section 1 is the introduction, Section 2 presents a review background, Section 3 describes a research methodology, Section 4 contains the findings and analysis of the selected primary studies based on the research questions, Section 5 presents the research discussion, Section 6 proposes a conceptual APT mitigation framework, Section 7 discusses the study limitations, and Section 8 provides the conclusion. 

## 2. Background

This section provides a concise summary of APT in Section 2.1, common device behavioral sources used for attack detection in Section 2.2, and APT mitigation approaches in Section 2.3.

### 2.1. Advanced Persistent Threat (APT)

This section presents a general overview of the formal definition of APT, its characteristics, and the APT attack process.

#### 2.1.1. A Formal Definition of Advanced Persistent Threat

APT initially referred to malicious, well-planned, and sophisticated cyber-attacks, whose goals originated from an external backer [24]. Specifically, an APT is a type of cyber threat that is malicious, well-organized, with hard-to-detect Tactics, Techniques, and Procedures (TTP), and it targets specific companies for long-term network access. Thus, in an attempt to obtain agreement on the definition of APT, the authors have provided a formal definition that is useful to researchers and practitioners: a malicious, coordinated, and highly-skilled entity that conducts a long-term or repetitive network infiltration and exploitation operation with the intent of obtaining information from a target organization, destroying its operations, or both [24]. As mentioned before, APT operations carry out one or both of the following primary functions: data collection or sabotage, i.e., capabilities that can be employed to deceive, degrade, disrupt, deny, destroy, or manipulate across the continuum [25,26].

#### 2.1.2. Characteristics of Advanced Persistent Threats

United States Air Force (USAF) analysts coined the term “Advanced Persistent Threat” in 2006 to make it easier to describe intrusive actions to their civilian counterparts [27]. Consequently, military teams could discuss the APT features without disclosing the identity of those who were engaged. The components of the term APT as coined by the USAF are as follows:

1. Advanced: The adversary is familiar with infiltration tools and may create its exploits [28];
2. Persistent: The adversary plans to carry out a task, get instructions, and achieve certain objectives [28];
3. Threat: The adversary is well-coordinated, well-supported, and well-motivated [28].

Because of their targeted nature, advanced attackers have intents and objectives that vary from those of traditional attacks. As shown in Table 1, some of the differences between APTs and traditional malware attacks are based on the APT features considered as the attack definition, attacker, target, purpose, and attack life cycle [29].

#### 2.1.3. Advanced Persistent Threat Process

Each APT campaign is unique in its behavior, and attacks are customized to a specific victim or organization [24]. Generally, in the APT attack process, after collecting the required information about the target, the first step is establishing a point of entry into the network [28]. Then, malicious software that is customized to a specific target creates a communication network that enables attackers to inject malicious code. In a stealthy fashion, this malicious software moves sideways through the system, sniffing for security vulnerabilities and exploiting them in order to infect other network systems. In addition, the malicious software creates copies of itself in order to preserve persistence inside the targeted system. As a result, APTs may establish new connections until they achieve their goal of either surveillance with data theft or disrupting the targeted system.

**Figure 1:**
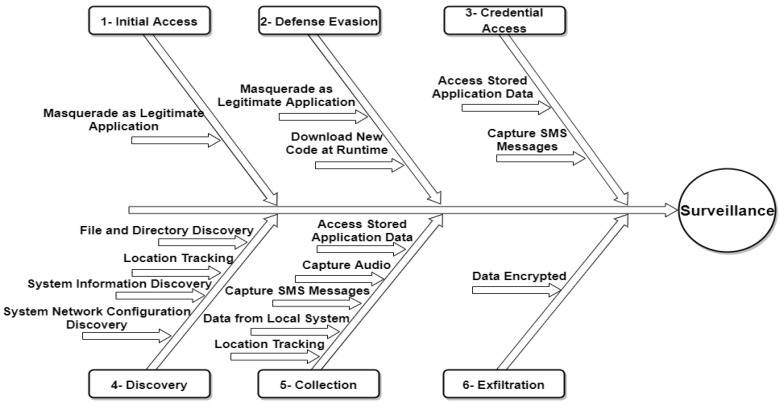
FrozenCell attack life cycle based on MITRE framework.

One example is the FrozenCell attack life cycle on mobile devices that has been described in the MITRE framework [30]. In the FrozenCell analysis, MITRE has presented the TTP of an APT attack, consisting of six stages (Tactics): (1) Initial Access, (2) Defense Evasion, (3) Credential Access, (4) Discovery, (5) Collection, (6) and Exfiltration [30]. Each of the stages between “Initial access” and “Exfiltration” does not have to take place in the same sequence every time. FrozenCell is a multi-platform attack called “Two-tailed Scorpion/APT-C-23” utilized to surveil the compromised mobile devices and desktop users [30]. The FrozenCell attack life cycle is illustrated in Figure 1 below.

1. Initial Access—The APT attack initially accesses the system using spear phishing with malicious executables that impersonate chat application updates such as Facebook, WhatsApp, and Messenger, in addition to applications that target Middle Eastern countries using the “Masquerade as Legitimate Application” technique;
2. Defense Evasion—After successfully accessing the targeted system, FrozenCell downloads and installs additional applications using the “Download New Code at Runtime” technique and establishes communication with a command and control (C&C) server controlled by APT attackers;
3. Credential Access—FrozenCell reads SMS messages and retrieves account information for other applications using “Access Stored Application Data and Capture SMS Messages” techniques;
4. Discovery—FrozenCell conducts a search about pdf, doc, docx, ppt, pptx, xls, and xlsx file types using the “File and Directory Discovery” technique. In addition, geolocation services for mobile towers are utilized by FrozenCell to track targets via the “Location Tracking” technique. Furthermore, FrozenCell captures the device manufacturer, model, and serial number, as well as phone information such as cell location, mobile country code (MCC), and mobile network code (MNC) using “System Information Discovery and System Network Configuration Discovery” techniques;
5. Collection—FrozenCell gathers the required information such as application account information, recorded calls, SMS messages, device images, and the location of the target;
6. Exfiltration—FrozenCell compresses and encrypts data before exfiltration by using password-protected 0.7z archives.

### 2.2. Common Device Behavioral Sources Used for Attack Detection

By 2025, 64 billion IoT devices will be connected to varied cutting-edge environments including smart cities, Industry 4.0, and crowdsensing (e.g., Flightradar24, OpenSky, ElectroSense) [31]. Because each of these environments has its own set of characteristics regarding devices, data, communication channels, and purposes, it is more difficult to meet their common challenges: optimizing device performance and providing an accurate service. To overcome these challenges, behavioral data science has evolved from studying theoretical and empirical issues regarding human behavior [32] to conquering the cyber world and providing a promising alternative to model device behaviors [33]. A device’s behavior could be classified as normal or abnormal based on how it operates [8].

**Figure 2:**
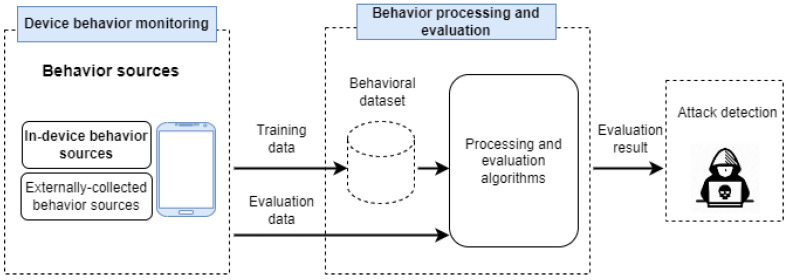
Common device behavior solutions life cycle.

In general, two main behavioral sources (external and in-device behavior) have been used to collect device behavior patterns in order to identify the suspicious activity that leads to abnormal device behavior [12]. Figure 2 illustrates the common device behavior solutions life cycle through three stages, including device behavior monitoring, behavior processing and evaluation, and APT detection.

#### 2.2.1. Device Behavior Monitoring

**Figure 3:**
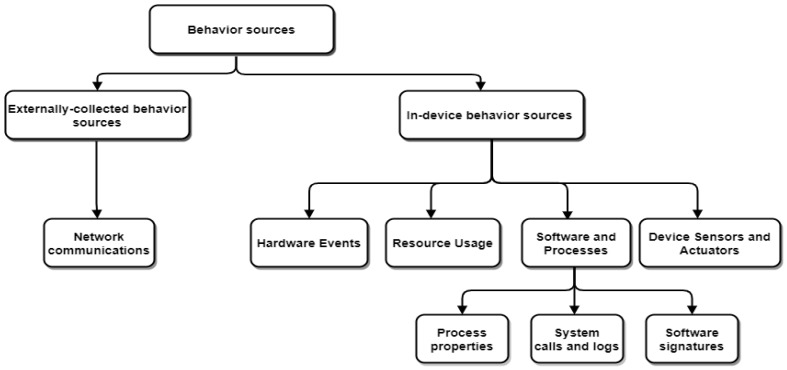
Behavior source classifications.

The first step is to monitor and collect the device behavior sources, which include in-device behavior and externally-collected behavior sources, as shown in Figure 3.

1. Externally-collected behavior sources—This category contains an external device (proxy or a gateway) that monitors devices and collects network-based data [12].
  - Network communications—From the perspective of the network’s communications, a diverse range of behavioral features can be collected from the network packets. These behavioral features rely on the traffic inspection granularity and the collected TCP/IP layers [34,35].
2. In-device behavior—In this category, the devices are subjected to behavioral data monitoring [12]. In the case of device behavior data, data is often gathered from different sources such as hardware events, resource usage, software and processes, device sensors, and actuators.
  - Hardware Events—In modern microprocessors, hardware performance counters (HPCs) are specific registers designated for storing hardware-related event counters. These events may be used to detect suspicious events [12];
  - Resource Usage—Device components’ use and status are monitored for anomaly detection. The most frequently observed components are the processor, memory, disk, and network [12];
  - Software and Processes—The installed software on each device has its own unique behavior. Then, in conjunction with the isolated software behaviors, a global device behavior may be modeled for anomaly detection [12]. Software may be modeled in a variety of ways, including:
    - System calls and logs—These features are used to observe the interaction between the operating system and its installed apps [36,37]. These interactions include activities for managing processes, files, and communications that have been utilized to detect abnormalities [36,37];
    - Process properties—The features of each process, such as its name, status, or threads, may be used to model the behavior of the device software. Resources needed to run specific software or code are also included in this category [38];
    - Software signatures—Software snapshots (signatures) may be used for the detection of software modifications caused by anomalous behavior [39].
  - Device Sensors and Actuators—These features, such as the camera, GPS, etc., may be used for anomaly detection [40,41].

#### 2.2.2. Behavior Processing and Evaluation Techniques

In the second step, to create and evaluate a fingerprinting profile, the data need to be processed using different approaches, including rule-based, statistical, knowledge-based, machine learning and deep learning, and time-series approaches [12].

To build and evaluate the performance of the learning model, the dataset is divided into two distinct sub-datasets. These two sub-datasets are the training data and test data [42]. Training data are the sub-dataset used to train a model. These datasets contain data observations in behavioral sources. While the test data are the sub-dataset used to evaluate the performance of a model built using a training dataset [42]. The purpose of creating a model is to predict known and unknown threats.

#### 2.2.3. Attack Detection

In the third step, detection may be achieved either by modeling normal device behavior and identifying abnormalities or by gathering normal and abnormal behavioral data and carrying out the classification methods in order to detect the suspicious activities [12]. Next, APT defense mechanisms will be present.

### 2.3. General Overview of Advanced Persistent Threat Mitigation Approaches

This section presents a general overview of APT mitigation approaches.

#### 2.3.1. Threat Modeling Approaches

A risk model can be defined as a quantitative depiction that identifies the threat possibilities and the impact they will have on a specific asset [43]. Threat modeling is a risk modeling component that identifies, prioritizes, monitors, and evaluates the security risks in an iterative process [43]. Threat modeling formalizes the process of identifying and evaluating the security vulnerabilities and threats of a device, an application, and a network service [44]. Threat modeling aims to be proactive in recognizing, categorizing, and describing threats that provide attacker visibility. This promotes resilience by preparing for, surviving, and recovering from a cybersecurity incident. The following is a list of the ten most important threat modeling approaches identified in this study:

1. DFD (data flow diagrams)—DFD is a graphical system depiction that illustrates all of the inputs, logical internal processes, and outputs. As part of the threat modeling process, DFDs focus on external elements and trust boundaries and storing and processing the data [45]. As a result of this method, the security analysts will be able to track data flow across the system in order to identify critical processes and threats to those processes. This approach has the following steps: view System as an adversary, characterize the system, and identify the threats [46].
  View System as an adversary analyzes the visible and accessible processes and functionalities that an attacker may use to breach the system. Characterizing the system means obtaining a background of system information and identifying weak points that need to be addressed. While identifying the threats includes thinking about and describing possible methods of attacking the entrance and exit points of the system [46];
2. STRIDE (Spoofing, Tampering, Repudiation, Denial of Service, and Elevation of Privilege)—STRIDE is a system-based threat classification that classifies threats according to their explicit types [47]. It was first introduced to Microsoft developers in 1999 to aid them in identifying threats related to their software products. The root cause might be classified as a security flaw in the design, a security bug in the code, or an issue resulting from an unsafe configuration [47]. STRIDE assists in mitigating risks regarding confidentiality, availability, authentication, authorization, and nonrepudiation [48]. STRIDE Categories may have several threats, or a threat can have multiple STRIDE Categories;
3. Attack trees—Attack trees are conceptual diagrams that utilize a branching, hierarchical data structure to represent threats and their possible attack vectors needed to achieve the attacker’s objective [49,50]. It was introduced by Bruce Schneier to represent threats against computer systems [43]. Attack trees categorize all known system attacks and assign risk and cost values to each attack vector [49]. Defining the main goal and breaking it down into sub-goals are common stages in the attack tree approach. The root node signifies the attack’s purpose, and the leaf nodes reflect the several paths that may be used to achieve that goal [51];
4. Stochastic or mathematical models—In this approach, attacks and their characteristics are often converted to Markov chains and analyzed using state transition matrices [52]. Markov chains have the ability to determine chains of attack vectors that require previous and current system states to be met before an attack may proceed on its current path [52].
  The game theory concept has also been used to model cyber threats such as APT. The game-theoretic basis is to build a multi-stage Bayesian game framework to capture incomplete information about deceptive APTs and their multi-stage movement [43];
5. Kill chain—The term kill chain originated as a military concept relating to the attack’s structure [43]. The idea is to effectively prevent or counter the opponent throughout the attack lifecycle [53]. The intrusion kill chain is defined as reconnaissance, weaponization, delivery, exploitation, installation, command, and control (C2), and actions on objectives (AOO) [53]. Effectively attributing cyber attacks requires identifying them based on their attack patterns and different phases of the kill chain. These attack patterns are Tactics, Techniques, and Procedures (TTP) of APT. A tactic is a behavior that is used to reach an objective, the technique is a potential method for implementing a tactic [54], and the procedure is a set of APT activities executed at each phase of the APT life cycle [55]. To achieve the APT’s goal, different tactics can be used. In turn, these tactics are accomplished by using one or many techniques;
6. MITRE ATT&CK—MITRE ATT&CK is an acronym for the Massachusetts Institute of Technology Research and Engineering, Adversarial Tactics, Techniques, and Common Knowledge [8]. MITRE established the Adversarial Tactics, Techniques, and Common Knowledge (ATT&CK) Framework in 2013 in an effort to better understand cyber threats [56]. MITRE had ATT&CK matrices associated with Enterprise assets (Linux/MacOS/Windows), mobile devices, and an initial PRE-ATT&CK pattern prior to October 2020 [43]. PRE-ATT&CK was a framework that aligns with the first three steps of the kill chain, namely reconnaissance, weaponization, and delivery. Version 11 of the ATT&CK Enterprise framework now includes PRE-ATT&CK and more closely aligns with all phases of the kill chain, including the post-access phases of exploitation, installation, C2, and AOO [43]. Tactics represent an adversary’s tactical objectives during an operation. The ATT&CK model’s techniques define the actions that adversaries may take to achieve their tactical goals. [57]. ATT&CK builds on the Cyber Kill Chain by concentrating on the techniques, tactics, and indicators of Compromise (IOC) associated with these adversaries. A significant difference between an ATT&CK technique and an IOC is that many ATT&CK techniques are legitimate system functions that may be utilized for malicious purposes [57], making them more difficult to detect by the defender. MITRE has also mapped software attacks from publicly reported technique use and accounts for the capability of the software adversary to use a technique [54];
7. Common Attack Pattern Enumeration and Classification (CAPEC)—CAPEC is a standard vulnerability database that provides a list of the most common methods attackers employ to exploit vulnerabilities identified in Common Weakness Enumerations (CWE) [43]. This means that CAPEC focuses on application security and defines the common characteristics and strategies used by attackers to exploit known vulnerabilities. CAPEC analyzes and categorizes cyber-attacks according to a set of attack patterns that may occur pre- or post-exploitation. In addition, it defines the stages of common cyber-attacks and documents their countermeasures. Within the CAPEC Model, there are three levels of the attack patterns (Meta, Standard, and Detailed) [43]. Attack patterns describe the characteristics and techniques used by adversaries to exploit known system vulnerabilities. The first is meta attack patterns, which lack detailed information on the technology or implementation by cyber attacks. The second is standard attack patterns, which are more procedural and specific. The third pattern is the detailed attack pattern.
8. Threat Assessment and Remediation Analysis (TARA)—TARA is a MITRE initiative that identifies and assesses cyber threats, as well as the effectiveness of countermeasures [58]. TARA includes an adversary TTP threat matrix called the Cyber Threat Susceptibility Analysis (CTSA). CTSA and Cyber Risk Remediation Analysis (CTRA) are then utilized to complete the TARA process [43]. CSTA consists of defining the assets in scope, identifying related TTP, removing unlikely TTP, applying a ranking system and constructing a threat matrix that defines the score, target assets, and adversary type [43];
9. Diamond—Diamond is a model that correlates and describes the capabilities of an adversary with the infrastructure of a target. It observes cyber-attacks assuming that the attacker’s targets and its TTP will vary over time [59]. The diamond threat model is a formal approach to applying scientific principles to intrusion analysis that maps the features of an adversary’s capacity to a target’s infrastructure [43]. It is used to track attack groups assuming that the attacker’s targets and its TTP will vary over time [59]. It derives its name from the diamond shape used to visually represent the four components of an intrusion: the adversary, the infrastructure, the capacity, and the victim. [59]. Similar to the Kill Chain and ATT&CK models, the diamond approach is based on an attacker using their (TTP) against a targeted system to achieve a predetermined objective. It provides a tested and repeatable approach for identifying activities and correlating them with an attack using quantifiable measures [43];
10. The National Institute of Standards and Technology (NIST) special publication 800-154—NIST 800-154 covers the fundamentals of threat modeling for data-centric systems [56]. Using NIST, threat modeling is described via a four-step qualitative approach [56]. The first step is the identification and characterizing stage that includes only specific information about a single system or a limited set of closely connected systems. The second stage, which is based on risk assessments, determines the possible attack vectors of an adversary (probability and effect). The third stage focuses on identifying security controls to mitigate particular attack actions. Finally, the threat model is analyzed to identify all possible attack vectors and security controls for unacceptably high risks [44].

#### 2.3.2. The Process of Risk Management Approaches

**Figure 4:**
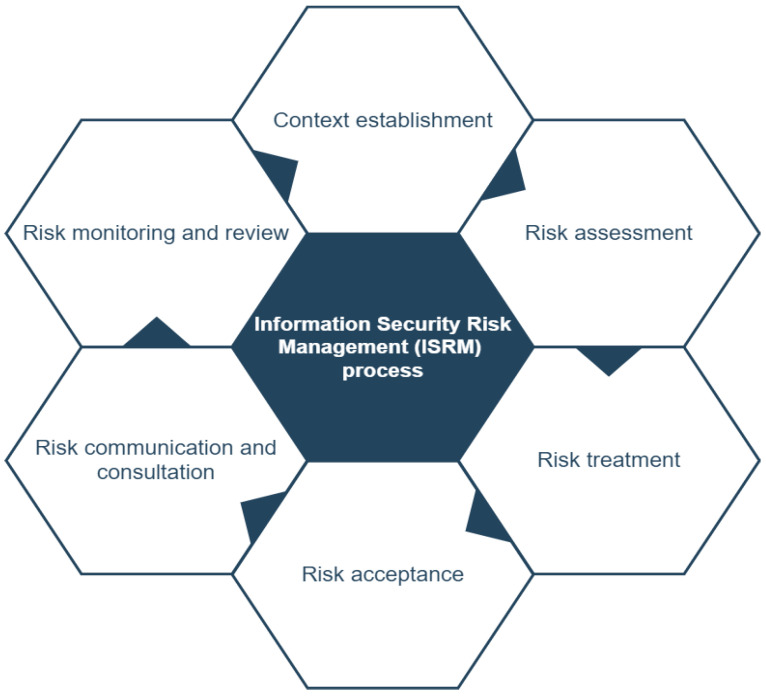
ISRM processes.

Along with the growing number of cyber-attacks, cybersecurity has grown to be one of the most vital parts of digital systems. The goal of cybersecurity is to decrease cybersecurity risks for organizations and users through the protection of digital assets and user privacy [60]. For such risks, a risk management system is required to identify risks and risk factors, as well as to propose approaches to decrease such risks [60]. One of the risk management models is Information Security Risk Management (ISRM). ISRM is the key means through which a business safeguards the Confidentiality, Integrity, and Availability (CIA) of the assets [61]. As illustrated in Figure 4, the ISRM process consists of the following steps:

1. Context establishment—The external and internal contexts for ISRM should be established, which includes identifying the fundamental criteria, defining the scope and bounds, and establishing an appropriate organization to operate the ISRM [62];
2. Risk assessment—This step necessitates gathering the required resource data (e.g., information assets, their vulnerabilities, mappings of each threat-asset-vulnerability combination, and identifying the possible effect of each risk scenario) [61]. The risk assessment process consists of three stages as follows:
  - Risk identification—Includes asset identification within the established scope, threat identification, control identification, and consequence identification of losses of CIA of the assets [62];
  - Risk analysis—In this step, the analysis of the risk is focused on the following: Consequence assessment (assess the potential information security incidents and their consequences that may result in the loss of CIA of an organization’s assets), Incident likelihood assessment (assess the possibility of a security incident), and Risk level determination (all relevant incident scenarios should have their own risk level) [62];
3. Risk treatment—Identify the security controls to decrease, preserve, avoid, or share risks, and define the risk treatment plan [62].
4. Risk acceptance—Make a decision to mitigate the risks to an acceptable level. The impact of this decision should be stated [62];
5. Risk communication and consultation—Decision-makers and other stakeholders in the decision-making process should exchange and/or share this risk information [62];
6. Risk monitoring and review—Risk factors (such as the asset value, effects, threats, vulnerabilities, and incident occurrence probability) should be observed and analyzed in order to determine the changes in the environment at an early stage [62].

#### 2.3.3. The Concept of Soft and Hard Trust Management

The trust concept has arisen for decades, if not centuries, in such fields as business, psychology, philosophy, and technology [63]. Trust in online social networks can be defined as users’ willingness to use those sites [64]. This is because a certain level of trust is needed to make the user willing to use the sites and share their private data on them. Trust management has been used to improve the security of networks by ensuring that a high degree of trust is maintained across network communications [65]. Soft trust and hard trust are the two main types of trust management that determine whether or not someone can be trusted [66]. Social control methods and intangible information, such as reputation, experiences, and collaboration, are used to establish soft trust [63]. In general, trust has many properties, such as the following:

1. Direct: In this feature, A and B have direct communication; the trust value is computed and inferred as a result of this direct communication [65];
2. Indirect: Trust is considered indirect when there is no direct connection between A and B. In order to determine the trust value of B, it is necessary to consider the recommendations that have been propagated to A from various nodes in the network [65];
3. Subjective: When trust is based only on a person’s own opinion, it is considered subjective [64,65];
4. Objective: If the trust is calculated based on specific parameters, such as the device’s quality of service (QoS), it is considered objective [65];
5. Local: The trust value between A and B is only valid between these two nodes. B may have a different trust value from another C in the network [65];
6. Global: A unique trust value is assigned to each node, which is known by all of the other nodes in the network [65];
7. Asymmetric: This means that even if node A trusts node B, node B may not trust node A in return [64,65];
8. History-dependent: In order to calculate trust, the nodes’ historical behavior is taken into consideration [65];
9. Context-dependent: The level of trust between A and B may vary from one context to another depending on specific events or conditions that have occurred between them [64,65];
10. Composite: The trust value may comprise a variety of factors such as honesty, reliability, security, etc. [65];
11. Dynamic: If any changes happen in the topology, the properties of the network, or the environment, the trust value should be updated accordingly [65].

However, soft trust is vulnerable to issues such as trust saturation: having a long history of positive experiences and cooperative efforts, a malicious entity such as an APT may accumulate high levels of trust in order to deceive the targeted entity (user and system) and successfully infiltrate the targeted system.

**Figure 5:**
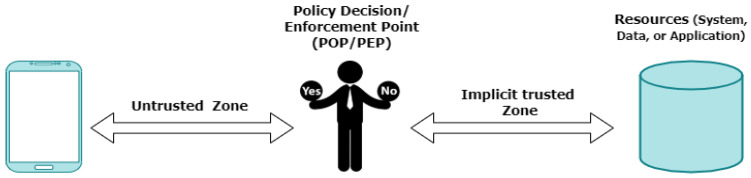
Zero trust resource access.

Hard trust, on the other hand, is generated from concrete security mechanisms and information, such as certificates and credential tokens [63]. One of the hard trust security mechanisms is zero trust. The concept of zero trust is based on the idea that organizations should never trust anything inside or outside of their perimeters [22]. Zero trust should verify anything and everything that is attempting to connect to the systems before it grants access [22]. Figure 5 depicts the abstract model of access with a policy decision point (PDP) and policy enforcement point (PEP).

Consider that when a smartphone user attempts to access a file on a network or server, the PEP describes the attributes of the user to other entities in the system. The PEP assigns to the PDP the task of determining whether or not a smartphone user should be authorized based on a description of their characteristics. The PDP analyzes policies that are stored on the system, makes its decision, and returns the decision to the PEP. The PEP then informs the smartphone user whether they have been granted access to the requested resource or not [22]. According to zero trust, the following five basic tenets are:

1. Access Segmentation—Each resource access needs to be properly segmented so that no single entity may access the whole/a large part of the network [67];
2. Universal Authentication—All entities that interact with the corporate network involving users, devices, applications, and workloads must be verified regardless of their network location [67];
3. Encrypt as Much as Possible—Zero trust considers the worst-case scenario, such as a data breach. This means that the network is constantly hostile, and thus trust cannot be automatically provided [67];
4. Least Privilege Principle—Each entity in a zero trust should be constrained to the minimum level of privileges to carry out a specific mission [67];
5. Continuous Monitoring and Adjusting—It is necessary to monitor each entity (internal or external) in a zero trust. This means that regardless of whether or not an access attempt is successful, all network traffic, system activities, and attempts to access the assets are observed and recorded [67].

#### 2.3.4. Situational Awareness Models

Cyber security has emerged as one of the most significant issues in today’s highly networked society. Situational awareness is a particularly prominent concept in the world of cyber security [68]. A cyber SA model is capable of monitoring and capturing different forms of threats, as well as analyzing and devising a plan to prevent further attacks [68]. Table 2 summarizes SA models that have been developed to provide quantitative indicators in decision-making [68]. First, Endsley’s model involves the observation of environmental factors within a certain time and space volume, the understanding of their meaning, and the projection of their future status [69]. Endsley’s model consists of three levels: perception, comprehension, and projection. The perception is a level that recognizes the status and attributes of related elements in the environment. The comprehension level is the step of synthesizing the elements of the perception level by analyzing and evaluating the situation. The projection level predicts how information analyzed at the comprehension level will affect the state of the future operating environment over time [70]. Beyod developed an Observe–Orient–Decide–Act (OODA) model that focuses on cognitive decision-making, as in Endsley’s model. OODA is a process that supports decision-making for dynamic environments [71].

Steinberg et al. studied the JDL Data Fusion Model (JDL DFM) combining processing, data fusion, and situational awareness. The JDL DFM consists of a structure that predicts and evaluates the monitoring environment depending on the information gathered in certain contexts. This model has the advantage of handling large amounts of data, such as network traffic [72]. Okolica et al. developed a cyber situational awareness model (CSAM) that reflects the company’s continuity plans. The CSAM model aims to build an automation engine that monitors the environment in real-time and predicts possible future risks based on sense, evaluation, and assessment [73]. Tadda and Salerno developed a situational awareness reference model (SARM) that combines Endsley’s model with the JDL DFM to improve data understanding. An advantage of this model is that it responds to ever-changing threats in real-time [74]. Evancich et al. studied effective cyber situational awareness (ECSA), which is situational awareness through network monitoring. ECSA is divided into three stages: network awareness, threat awareness, and operational awareness. The network awareness stage is to identify the network security characteristics. The threat awareness stage is to identify possible attacks and their attack vectors. The operational awareness stage is to measure the attack’s impact on the network [75].

## 3. Research Methodology

The research methodology has been utilized to explore device behavior-based APT defensive mechanisms. An SLR requires understanding, assessing, and determining the research evidence to address specific review questions [76].

### 3.1. Review Questions

The purpose of the research question is to assess and review the existing studies. Population, Intervention, Comparison, Outcomes, and Context (PICOC) criteria have been used to formulate these questions [77] as shown in Table 3.

The research questions are as follows:

RQ1—What are the APT activities reported by researchers?
RQ2—What are the proposed defensive mechanisms available to defend against APT?
RQ3—What are the existing risk management techniques utilized by the primary studies?

### 3.2. Review Protocol

The search process includes selecting the digital repositories, constructing a search string, conducting an initial search, and retrieving the first primary studies collection. Five digital repositories have been utilized in many SLRs [78]: Springer Link, Science Direct, Association for Computer Machinery (ACM), Scopus, and IEEE Xplore. Following the digital repository selection, a search string was necessary to conduct a comprehensive search and choose the related primary studies. To define a search string, the following four steps should be taken:

1. Formulate the research questions based on PICOC criteria to define the main keywords;
2. Recognize synonyms and other spelling variations for each main keyword;
3. Verify search keywords included in titles, abstracts, and keywords;
4. Construct a search string using the Boolean conjunction operators.

The following search string was selected by an independent panel of experts: (“Advanced persistent threat*” OR “APT”) AND (“Mobile” OR “Smartphone” OR “Internet of things” OR “Internet-of-things” OR “IoT” OR “computer*”).

This search string was used to gather all available primary studies in the five digital libraries. To choose the related studies from the initial list, inclusion and exclusion criteria were created.

Inclusion criteria:

- Papers are written in the English language;
- Published from 2011 to 2022;
- Published in a journal.

Exclusion criteria:

- Articles are written in a language other than English;
- Papers that do not refer to research questions or do not adequately identify the subject;
- Research papers of less than three pages.

**Figure 6:**
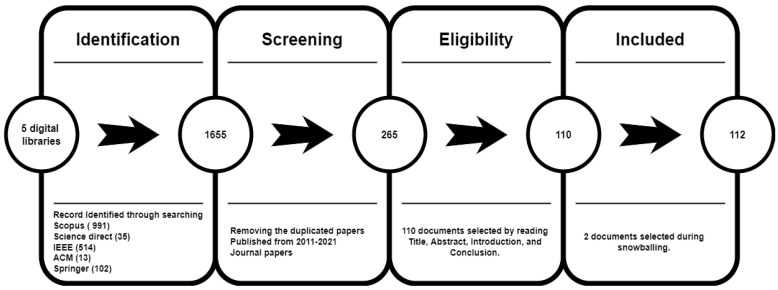
PRISMA flowchart for relevant paper selection.

As illustrated in Figure 6, the selection process was divided into four stages, as follows:

- Identification: The search string was performed on five digital libraries: Springer Link, Science Direct, Association for Computer Machinery (ACM), Scopus, and IEEE Xplore and 1652 papers were retrieved.
- Screening: After eliminating duplicated papers in the last twelve years (2011–2022), non-English language papers, and non-journal papers, the authors were left with 265 papers.
- Eligibility: Related papers were identified by searching title abstracts and keywords in the digital libraries. Papers with inadequate information to answer the research questions were excluded. The selected papers were further investigated by reading each one’s introduction and conclusion. Papers deemed irrelevant were eliminated.

In the end, 110 journal papers were selected. Forward and backward snowballing was also used (this involves looking to see if any other relevant papers were published after the chosen one and citing the chosen one). As a result, only journal papers published between 2011 and 2022 were included in the study.

- Included: In this criteria, two new related papers were identified, thanks to snowballing. As a result, 112 journal papers were selected.

## 4. Analysis and Findings of Research Questions

In this study, the primary studies that were utilized to answer the research questions were retrieved from five digital repositories. Table 4 summarizes the APT features with information on them and on ATT&CK. APTs are multi-stage attacks that use different techniques to accomplish their goal. These attacks have been viewed in stages using threat modeling, such as the MITRE framework. Table 5 contains APT defensive mechanisms and provides information on the technique, components, platform, and APT defense mechanisms. These APT defensive mechanisms are classified into artificial intelligence (AI), machine and deep learning, game theory, situational awareness, risk management, trust management, and access control. Table 6 summarizes risk management approaches for APT mitigation with information on the approach, platform, and attack type. Next, we will present the findings and analysis of the research questions.

### 4.1. RQ1: What Are the APT Activities Reported by Researchers?

In this section, the findings and analysis of Research Question 1 related to APT features are presented. APT is a hard-to-detect cyber threat group or campaign that may use familiar attacks (such as spear phishing, watering hole, application repackaging, etc.) but the tools utilized are advanced, stealthy, and sophisticated, which could avoid the defense mechanisms.

**Figure 7:**
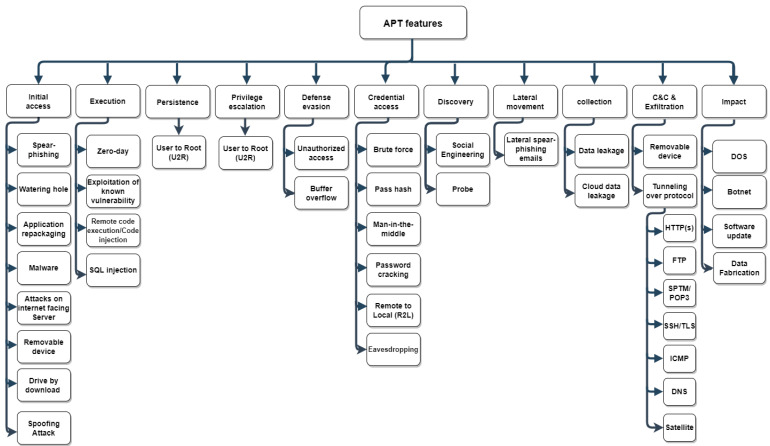
ATT&CK-based taxonomy of APT features.

These APT features have been grouped based on the APT life cycle using threat modeling approaches such as MITRE, ATT&CK, and Cyber Kill Chain [43]. Specifically, the MITRE framework is used to classify APT attacks according to their tactics and techniques. The ATT&CK-based taxonomy is shown in Figure 7, and Table 4 depicts the mapping between the collected APT features and the ATT&CK-based taxonomy. The ATT&CK matrix consists of eleven tactics (from Initial Access to Impact) as follows:

#### 4.1.1. Initial Access

The Initial Access stage comprises malware delivery using common direct delivery methods such as social engineering [171]. In this stage, several techniques employed by APT to compromise the target system include:

1. Spear phishing—The attacker attempts to induce the victim to click a malicious file, application, or web link in order to successfully infiltrate the targeted system [1,3,7,28,79,80,81,82,83,84,85,86,87,88,89,90,91,92,93,94,95,96,97,98,99,100].
2. Watering hole attack—A watering hole attack is a type of cyber-attack that focuses on a specific group of people by infecting websites they often visit [3,28,79,84,88,99,101,102];
3. Application repackaging—The attackers modify a popular application that has been downloaded from application markets, reverse engineer the application, inject malicious payloads into the application, and then release the modified application [1,3,88,89,102,103,104,105];
4. Malware–Malicious software such as spyware, Trojans, and bots are used to carry out unauthorized operations on a targeted system in order to steal information or disrupt the system [87,116,121,144,172,173,174];
5. Drive-by download—A user visits a website, opens an email attachment with a malicious file or weblink, or clicks on a misleading pop-up window that prompts the user to download malware [3,89,107];
6. Infected storage media—Malware-infected USB devices or CDs/DVDs have been used to locally deliver malicious software to the targeted systems [3,83,89,101];
7. Attacks on Internet-facing servers—Access to the target’s internal infrastructure is established through penetrating Internet-facing servers. To penetrate these servers, credentials are often obtained using brute-force attacks or exploiting known server vulnerabilities [106];
8. Spoofing attack—Attackers appear to be someone or something else in order to gain the confidence of the targeted user and gain access to systems [96].

#### 4.1.2. Execution

This tactic involves injecting adversary-controlled code into a program, either remotely or locally. Malicious code execution techniques are frequently used with other techniques to achieve broader goals, such as network discovery or data theft [175]. Based on the literature, four types of APT attacks are utilized in this stage, as follows:

1. Zero-day exploit—This attack takes advantage of an undiscovered software vulnerability for which no updates or fixes are available [3,5,82,84,86,87,88,90,94,97,101,109,110,111,112,113,114];
2. Known vulnerabilities exploit—Some cyber-attacks make use of exploit kits to gain access to a target network by exploiting known vulnerabilities that have been left unpatched [87,114];
3. Code injection—Also referred to as remote code execution (RCE), this occurs when an adversary takes advantage of a software input validation vulnerability to inject and perform malicious code [79,101,115];
4. SQL injection—Code injection techniques are used to mislead the database server into executing malicious SQL codes that have been injected into the database [7,82,108].

#### 4.1.3. Persistence

The attackers seek to maintain their foothold through each access, action, or configuration change to the targeted devices [176]. A User to Root (U2R) attack is used to maintain the foothold by gaining root access to the target system [116].

#### 4.1.4. Privilege Escalation

An attacker’s ability to get more privileges is known as privilege escalation. The attacker may utilize the newly gained account privileges to potentially gain full control of the targeted system and perform lateral movement in the network [177]. One attack in the privilege escalation stage is U2R.

1. User to Root (U2R)—U2R attacks happen when the attackers successfully compromise a normal user’s account and escalate their privileges to get root access to the target system [116].

#### 4.1.5. Defense Evasion

Defense evasion refers to the strategies that an attacker may use in order to circumvent defense mechanisms [122]. For example, an attacker might exploit vulnerable components of a web application to circumvent security controls and get access to a database [171]. Two examples of APT attacks that may be used are unauthorized access and buffer overflow.

1. Unauthorized access—This type of attack occurs when a person gets access to a digital system without the consent of the user [6];
2. Buffer overflow—This is a coding error or vulnerability in software that attackers may take advantage of in order to obtain unauthorized access to targeted devices [108].

#### 4.1.6. Credential Access

An attacker may use credential access such as passwords, tokens, cryptographic keys, or other values to gain access to resources [123]. Various attacks may be employed to steal credentials from the targeted devices, as follows:

1. Brute-force attack—This occurs when an attacker submits a large number of passwords or passphrases in the expectation of guessing correctly eventually [28];
2. Pass-the-Hash (PtH)—An attacker captures a hash of a password instead of the password characters and then uses it to authenticate and possibly get access to other networked systems [28];
3. Man in the middle (MITM)—Communications between two parties are eavesdropped on to collect login credentials or personal information, spy on victims, disrupt communications, or cause data to be corrupted, among other purposes [79,82,83,117,118];
4. Password cracking—The attacker may run a password cracker or purchase a password in an underground forum [119];
5. Eavesdropping attack—This is also referred to as a sniffing or snooping attack. Passwords, credit card information, and other sensitive data are easily stolen during the transmission of data from one device to another [120].

#### 4.1.7. Discovery

The Discovery tactic includes techniques such as social engineering and probing attacks to enable the adversary to gather information about the targeted system’s features and potentially other networked systems [127].

1. Social engineering—In order to obtain information and gain access to a system, social engineering attacks often target people as their primary target. Most APT attackers use this technique to gather information about the targeted user at the reconnaissance stage, moving laterally to other systems or figuring out the compromised systems [78,80,81,82,85,87,97,105,107,111,121,122,123];
2. Probing attack—This is a passive attack that relies on methods such as footprinting and social engineering to gather information about a particular system [124].

#### 4.1.8. Lateral Movement

The attacker attempts to gain access to additional services on the target system or network [171]. The attackers aim to get authentic credentials that will enable them to remain in the system by using different techniques such as lateral spear-phishing emails [100,125].

#### 4.1.9. Collection

At the collection stage, the attacker attempts to obtain the data of interest [171] using different techniques such as data leakage/cloud data leakage attacks.

1. Data leakage—This attack happens when a source (a person or a device) within the business sends data to an unauthorized entity (the attacker) outside the organization without permission [108].
2. Cloud data leakage—This attack happens when the attacker is trying to disclose information about an organization’s customers or the services it provides without the organization’s consent [108].

#### 4.1.10. Command and Control

In the command and control (C&C or C2) stage, the attacker is trying to communicate with the compromised systems within a target network [129]. The adversary can establish C&C through either network protocols or removable media.

1. Network protocols—For remote connection and data transfer, most C2s utilize the standard Hypertext Transfer Protocol (HTTP) or other common network protocols such as the File Transfer Protocol (FTP), the Simple Mail Transfer Protocol (SMTP)/Post Office Protocol (POP3), the Secure Shell (SSH)/Transport Layer Security (TLS), the Internet Control Message Protocol (ICMP), the Domain Network System (DNS), or other network protocols [100,126];
2. Removable media—Attackers may misuse removable media, such as a USB drive or a hard disk, to transmit malicious files or exfiltrate data [126].

#### 4.1.11. Impact

The attackers are attempting to manipulate, interrupt, or even damage both the devices and the data they are collecting [178]. At this stage, different techniques are used by the attacker to execute the mission objectives, as follows:

1. Denial-of-Service (DoS)—A DoS attack damages the compromised system or network and renders it completely inoperable, making it unavailable to its intended users [3,76,79,81,92,97,111,115,124,125,126,127,128,129,130];
2. Botnets—Botnets are groups of Internet-connected devices (remote sensors), each of which is running one or more bots that may be used for a variety of purposes, including DoS, information theft, and SPAM spreading [4,82,131];
3. Software Update Attacks—Software update attacks may be used to compromise system integrity and availability by disrupting the updating process of the installed software [108];
4. Data Fabrication—Data fabrication is the generation of malicious data or processes in order to exploit access granted for a different reason, such as tampering with system integrity [108].

According to the findings of RQ1, APT features can be viewed through stages using threat modeling frameworks. One of the threat modeling frameworks is MITRE, which groups APT attacks based on their tactics and techniques to describe the characteristics of the attacks. As a result, an APT utilizes sophisticated and advanced techniques to exploit the known and unknown system vulnerabilities and successfully infiltrate the targeted devices. An APT has the capability to remain stealthy by avoiding detection techniques for a long period. In addition, APT utilizes different discovery techniques to achieve its goal, whether it is espionage with data theft or disrupting the systems. Next, we will present the analysis and findings of RQ2.

### 4.2. RQ2: What Are the Proposed Defensive Mechanisms Available to Defend against APT?

In this section, the findings and analysis of RQ2 related to APT defense mechanisms are presented. A general insight of defense mechanisms against APTs on different platforms such as computers, IoT, and mobile devices is presented. The main purpose to do such a classification is to categorize the impact of APTs based on different platforms and to analyze the contribution of the primary studies on mobile APTs with other platforms. This means that there is a lack of contribution to defending against mobile APTs.

This study identified 60 primary studies of which 40 primary studies focused on APT detection [1,3,5,6,7,28,76,86,97,98,100,106,111,115,124,125,126,127,128,129,134,136,137,138,139,142,172,173,176], six primary studies focused on APT protection [4,91,92,118,121,125], four primary studies focused on APT mitigation [142,143,144,145], three primary studies focused on APT identification [98,107,146], and [116] focused on the detection and response to APTs as shown in Table 5.

**Figure 8:**
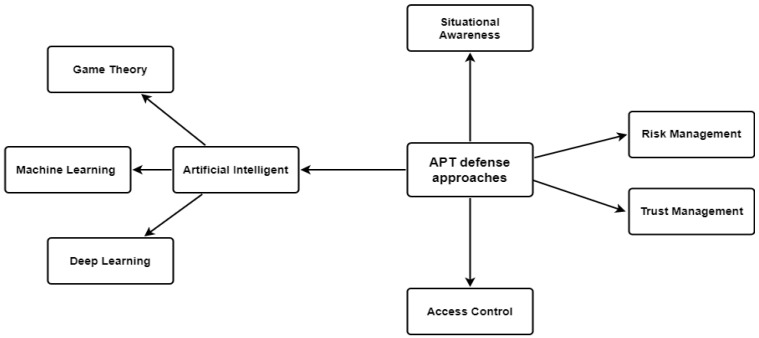
APT defense approaches.

As illustrated in Table 5 and Figure 8, many APT security defense mechanisms have been invented to protect a system’s security, such as game theory, access control, risk and trust management, artificial intelligence, and machine and deep learning techniques.

One of the most common AI detection solutions used in the literature is AI techniques. Many AI techniques involving machine learning (ML) and deep learning (DL) that have been proposed by various researchers are either network-centric [1,3,6,7,79,82,83,84,90,91,92,93,103,107,111,112,113,116,118,121,125,130,131,133,134,135,136,137,139,140,141,142,143,144,145,146,147,148,149,150,151,152], device behavior-centric [105,109,138], application-centric [5,86,110,124], or network and device-centric [89,117]. However, current network-based detection systems are ineffective against APTs because APTs employ sophisticated techniques such as encrypting the payload or using a secure communication such as SSL (e.g., Cloud Atlas APT). Device behavior models [105,109,138] fail to tackle an APT issue using system behavior models because they fail to map the behavior to the unique characteristics of APT attacks [17]. Malware spreads via custom encrypted partitions on removable media (e.g., ProjectSauron APT) and exploits weak points in authentication mechanisms [117].

Furthermore, with the application-centric detection systems [5,86,110,124], the malware characteristics are generally categorized into static features (such as binary file characteristics and disassembly features) and dynamic features such as execution behavior features [179]. Static features may be difficult to extract because of APT attacks’ polymorphism, distortion, and shelling. Dynamic features are often collected by monitoring the program’s behavior at runtime, which may be affected via confusion technology [179].

In addition, APTs can be tackled using game theory. Game theory techniques have been utilized to detect or mitigate APTs on IoT [1,82], computers [98,145], and in general [135,151,152]. In addition, game theory has been used with risk management approaches to identify the APT in fog computing [111] and IoT [107]. Furthermore, it has been used with trust management techniques to protect cyber-physical systems [92] and IoT [116]. While game-theoretic models can help understand attacker behaviors and incentives, these models are founded on certain assumptions, such as unbounded rationality on the part of players, which may not be realistic or have a limited input data [180].

Furthermore, risk management approaches are a second solution to manage the risk caused by APT. The primary studies have focused on identifying APTs using risk management [93,132,146]. However, according to [61], there are endemic deficiencies in managing risk: (1) The identification of information security risks is often a tedious task; (2) Information security risks are often calculated with little reference to the actual situation of the organization; and (3) Risk assessments for information security are often conducted on an intermittent and non-historical basis. As a result, risk management approaches inevitably lead to poor decision-making and inadequate or inappropriate security strategies to protect the user’s data.

In addition, trust management approaches are a third solution to authenticate the resource requested by the user. Trust management approaches have been proposed to protect cloud computing [91]. However, soft trust is vulnerable to issues such as trust saturation: having a long history of positive experience and cooperative efforts, a malicious entity such as an APT may accumulate high levels of trust in order to deceive the targeted entity (user and system) and successfully infiltrate the targeted system. Two primary studies have used access control approaches to protect APTs on mobiles [102] and IoT [4]. However, these models are mathematical models and are not implemented in real-world deployments.

Finally, decision-making models are the other solutions that have been proposed to monitor and capture different kinds of threats, and analyze and create a plan to mitigate further threats. The Endsley situational awareness model has been used to detect APT attacks on IoT [113,141]. However, these primary studies introduced only one stage of the three stages of the SA model (perception, comprehension, and projection).

According to the findings of RQ2, the authors classify the APT defense mechanisms into five techniques that include situational awareness, risk management, trust management, access control, and artificial intelligence. Based on the literature, most APT defense solutions are AI techniques, most of which are network-centric while the others are device-centric. The finding and analysis of RQ3 will be presented next.

### 4.3. RQ3: What Are the Existing Risk Management Approaches Utilized by the Primary Studies?

This section presents a general overview of risk management approaches against cyber-attacks. This study identified 23 primary studies, of which two focused on mobile [153,154], six focused on IoT devices [107,155,156,157,158,181], two focused on cyber-physical systems [168,169], four focused on either fog computing [111,160], the 5G edge-cloud ecosystem [167], or connected and autonomous vehicle (CAV) [159], and eight in general [61,93,161,162,163,164,165,166]. Furthermore, four primary studies focused on APT [93,107,111,160], while the others are focused on either specific attacks such as DDOS attacks [181], DOS [167], SQL injections attacks [181], or privilege-induced attacks [154], or non-specific attacks [60,61,140,155,156,157,158,159,161,162,163,164,165,166,167,168,169], as shown in Table 6.

**Figure 9:**
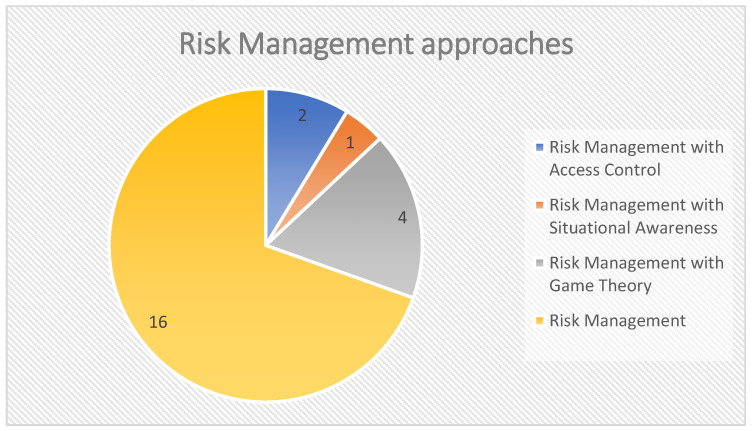
Risk management approaches.

As shown in Figure 9, the existing studies either proposed risk management approaches [93,154,155,156,158,159,161,165,166,167,168,169] or were integrated with other approaches such as access control [161,164], situational awareness [61], and game theory [107,111,160,162].

Many researchers have proposed different risk management approaches to minimize the threats and risks to IoT [158,181], computers [170], cyber-physical systems (CPS) [168,169], the 5G edge-cloud ecosystem [167], connected and autonomous vehicles (CAV) [159], and others [89,156,160,169]. Other researchers [154,155,156] have proposed guided frameworks that aim to support practitioners to formulate or reframe their IoT security risk management strategies.

In addition, risk management can be used with other approaches such as access control, SA, and game theory to support the decision-making process. Based on the work done by [153,164], the authors proposed risk management with access control to support the decision-making process to recognize the risks and their attributes from the monitored environment. Furthermore, a conceptual situation-aware ISRM (SA-ISRM) model complements information security risk management to address an enterprise-wide collection, analysis, and reporting of risk-related information [61]. On the other hand, risk management could be used with game theories to minimize cyber risks. The authors proposed a game theory for cyber risk management to design cyber insurance contracts to transfer the cyber risk from either fog computing [111,160,162] or IoT [107].

Based on the findings of RQ3, the authors classify primary studies into four categories: risk management with access control, situational awareness, game theory, and risk management. Of these, most of the primary studies have focused on traditional attacks, while only three primary studies have focused on APT. Furthermore, many existing studies have focused on qualitative approaches due to their simplicity, risk appetite, and ability to evaluate risk. The problem with qualitative methods is that they are subjective and imprecise. Next, the research discussion will be presented.

## 5. Research Discussion

In this SLR, the authors have reviewed 109 journal papers on APT attack–defense mechanisms that were published from 2012 to 2022. All available journal papers have been collected from various digital libraries such as Springer Link, Science Direct, Association for Computer Machinery (ACM), Scopus, and IEEE Xplore. The authors have provided a summary of APT features, APT defense mechanisms, and a general overview of the risk management approaches that have been proposed to identify these APT features. Next, the research gap and recommendations for future investigations will be presented in Section 5.1 and Section 5.2, respectively.

### 5.1. Research Gaps

This section presents the research gaps in the existing APT defense solutions. Following the existing APT defense solutions defined above in Section 4.2, the authors present the following research gaps:

#### 5.1.1. Solution Techniques Are Ineffective and Not Fully Bullet-Proof

Most of the APT defense solutions [1,3,4,5,6,7,28,76,86,87,88,94,97,98,100,101,102,103,106,108,111,115,117,121,124,127,133,134,135,136,140,141,142,143,145,147,172,173,176,178] being investigated have loopholes and limitations. Based on the literature, the APT defense solutions have focused on identifying, protecting, detecting, and responding to APT attacks. The most widely used techniques to detect APT attacks are machine and deep learning [3,5,7,28,79,80,81,82,84,85,86,97,111,115,124,125,126,127,128,129,134,136,138,172,173,176]. However, these techniques are not capable of detecting an improved or unknown APT malware due to the ever-increasing and changing threat scenarios posed by it, e.g., ZooPark [9]. This ever-changing threat landscape leads to a lack of a clear and comprehensive understanding of the TTP of APTs [23]. Other solutions proposed risk management approaches that focused on APTs [93,132,146] or traditional attacks [93,111,161,162,163,164,165,166,167,168,169,170]. Most of the existing studies have focused on qualitative approaches due to their simplicity, risk appetite, and ability to evaluate risk. The problem with qualitative methods is that they are subjective and imprecise [168]. Furthermore, risk management solutions have endemic deficiencies in managing risk: (1) The identification of information security risks is often a tedious task; (2) information security risks are often calculated with little reference to the actual situation of the organization; and (3) risk assessments for information security are often conducted on an intermittent and a non-historical basis [124]. As a consequence, poor decision-making and insufficient or incorrect security techniques to safeguard the user’s data are the outcomes [61]. Other solutions include protecting digital systems against APTs using trust management [91] or access control [4,102]. The trust management solution is ineffective in detecting APTs as a soft trust and is vulnerable to issues such as trust saturation: having a long history of positive experience and cooperative efforts, a malicious entity such as an APT may accumulate high levels of trust in order to deceive the targeted entity (user and system) and successfully infiltrate the targeted system. The limitations of the access control solutions are a lack of exploration of the human behavioral context in terms of their intention, device usage, and tasks done with a smartphone. Finally, mitigating malicious network traffic as a response to incidents is another solution [116]. However, this solution is not effectively designed to detect and prevent only known attacks, as APTs use sophisticated methods such as encrypting the payload or using a secure channel via the SSL protocol (e.g., Cloud Atlas APT), and exploiting vulnerabilities in authentication mechanisms [111].

#### 5.1.2. Solution Techniques Are Unable to Detect APTs in a Timeframe

Some of the APT defense solutions [3,5,7,28,80,81,82,84,85,86,97,111,115,124,125,126,127,128,129,134,136,138,171,172,173,176] may not be capable of the early detection of APTs. APTs have the ability to easily avoid digital-signature-based and anomaly-based defense techniques and attempt to gain long-term access to the targeted systems. The detection of such APTs could take months or even years. The prime example, Stuxnet, which has targeted programmable logic controllers (PLCs) of sensitive industrial systems, was active for at least three years until its discovery [15]. The other example is ZooPark, a cyberespionage toolkit that targeted Android devices in 2015 and was active for three years until its discovery in 2018 [9].

#### 5.1.3. Attack Paths Are Unclear and Proprietary to Models

**Figure 10:**
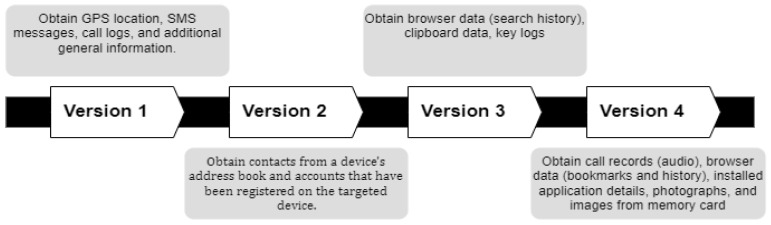
ZooPark attack versions.

An APT attack is hard to mitigate due to its non-deterministic fingerprint or TTP. Various frameworks such as Cyber Kill Chain and MITRE collect different TTPs for the same APT attack. For example, the APT 28 life cycle in the Cyber Kill Chain consists of seven stages [182], while the APT 28 life cycle in the MITRE framework consists of 14 stages [183]. In addition, APT groups have evolved and are continuing to extend their existing targets, necessitating the implementation of new TTPs [3]. These attack groups are capable of developing malware and data exfiltration techniques that are well suited for their intended goal [3], as shown in Figure 10.

Currently, ZooPark is still active [9] and ZooPark malware has been found in four variants by security experts. In the original ZooPark attack, only a small amount of sensitive information was acquired from the targeted systems. However, as the attack evolved, the malware’s capabilities grew and the attackers were able to collect almost any information they wanted.

#### 5.1.4. Existing APT Device Behavior Solutions Fail to Solve the APT Issue

Based on previous studies, most of the solutions [13,14,15,16,17] have failed to tackle an APT issue using system behavior models because the existing detection studies fail to map the behavior to the unique characteristics of APT attacks for the following reasons: Some of the APT solutions are lacking APT detection for every stage of the attack life cycle. Work done by Mohammad and Belaton [13] focused on the credential dumping technique through monitoring CPU, RAM, windows registry, and file systems in order to detect APT. However, the authors only focused on one stage of the APT (the credential access stage) and did not provide a comprehensive solution to detect APTs in all stages of the APT life cycle. Other APT detection solutions such as [15,16,17] proposed IDS to model the device behavior in order to detect APTs using system events. However, these techniques may raise false-positive alarms when normal system behavior changes, or may not be able of the early detection of APT.

#### 5.1.5. Biased Solutions in Terms of Grouping

Most current APT defense solutions such as detection solutions [1,3,5,6,7,28,76,86,97,98,100,106,111,115,124,125,126,127,129,134,136,137,138,139,142,172,173,176], identification solutions [94,121,133], protection solutions [4,13,91,92,118,121], and mitigation solutions [116] are generalized based on a group of users instead of a single individual protection [184]. These solutions result is bias because they are used to protecting groups of users and cannot be assumed to be accurate for individual protection, as users often have many other confounding variables that impact their behavior [185]. This means the risk of each device’s behavior varies according to the user’s behavior [18].

### 5.2. Recommendations for Future Investigations

In this section we provide recommendations for future investigations to design a model that has the capability to overcome these research gaps in Section 5.1.

#### 5.2.1. To Design an Effective Solution That Follows a Cyber-Security Framework Such as NIST or ISO

A cyber security framework is a risk-based approach to managing cybersecurity risk [186]. Based on Clark Nuber PS [187], one of the cyber security frameworks to implement and improve the cyber security Program is NIST [20,187]. NIST categorizes the cybersecurity capabilities into five core functions (Identify, Protect, Detect, Respond, and Recovery) [20]. One of the identification solutions is risk assessment. Skipping this step tends to over-secure the environment, resulting in lost resources [187]. Risk assessment includes identifying the asset in order to secure personally identifiable information (PII) and cyber threats to these assets such as APT. In the protection stage, the authors recommend utilizing the zero trust model. This model is used to prevent the increasingly severe risk of data leakage [21] and lateral movement [22]. One of the core tenants of the zero trust model is universal authentication. This means that all entities, including users, devices, applications, and workloads, having any form of interaction with the corporate network, need to be authenticated regardless of their network location [73]. In addition, a Host Intrusion Detection System (HIDS) is recommended at the detection stage to detect any suspicious activity. At this stage, risk assessment is used to assess the likelihood and impact of the risk by quantifying the device’s behavior and its components such as (CPU, memory, battery, network (sent and received data)) and user activity. For the response stage, the authors recommend utilizing risk mitigation approaches. Risk mitigation is the second process of risk management that is used to reduce mission risks such as risk assumption, risk avoidance, risk limitation, risk planning, research and acknowledgment, or risk transference [186]. Finally, at the recovery stage, data backup and recovery techniques have been used for APT incident recovery [188].

#### 5.2.2. To Design an Efficient Solution That Has a Decision-Making Model Using Cyber SA

According to Andrade and Yoo [189], there is a need for a cognitive security model that integrates technological solutions such as big data, AI, and support decision systems with the cognitive processes of security analysts used to generate knowledge, understanding, and execution of security response actions. A cognitive security model can help security analysts to make precise decisions in detecting suspicious incidents in less time and more efficiently. A cognitive security model such as Cyber-Cognitive Situation Awareness (CCSA) is self-aware and is capable of acquiring the following three properties at execution time: (1) Auto-reflective: It is aware of its software architecture, hardware infrastructure, and execution environment in order to meet its operational goals, (2) Auto-predictive: It is capable of predicting the effects of a dynamic change caused by potential adaptive actions, and (3) Self-adaptive: It has the ability to meet its operating goals despite changes in the environment [189].

**Figure 11:**
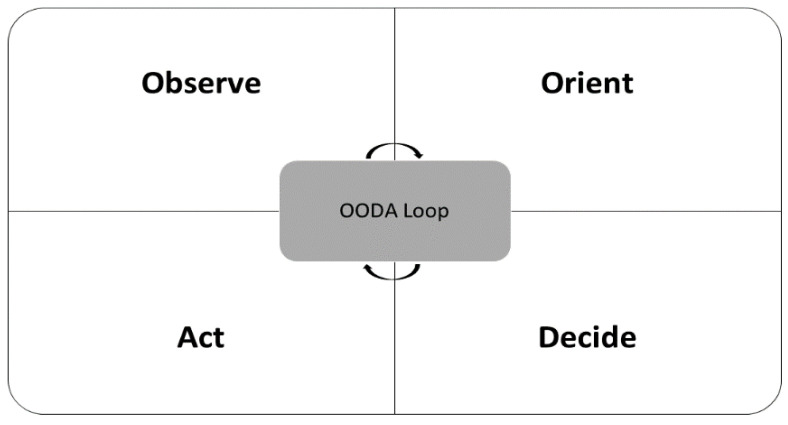
OODA loop.

CCSA has the ability to monitor and capture different kinds of threats, as well as analyze and devise a strategy to prevent further threats [68]. One of the SA models is the Observe–Orient–Decide–Act (OODA) model. OODA’s goal is to overcome the APT detection issue and raise surrounding environmental awareness. Figure 11 shows the four phases of the decision-making cycle [190]. In an OODA loop, each phase represents a process that is in constant communication with its environment. Observation is the process of monitoring and gathering environmental data [190].

It is guided and controlled by the Orient phase while receiving feedback from the Decide and Act phases. The Orient phase is the process of analyzing the data gathered in the Observation phase, taking into consideration the potential Orient phases from previous loops [190]. It is possible to eliminate unnecessary data by looking for correlations and dependencies that may be employed in the decision-making process. The Decide phase determines which hypothesis will be performed depending on the environment context [190]. It is guided by the Orient phase’s input and provides feedback to the Observe phase. Finally, in the Act phase, the specified hypotheses are put to the test by interacting with the surrounding environment [190]. It is guided and controlled by the Orient phase, receives feedback from the Decide phase, and provides feedback to the Observe phase.

#### 5.2.3. To Design Attack Paths Using Threat Modeling Approaches

Based on Sanchez et al. [12], one of the most promising approaches to dealing with APT issues is device behavior fingerprinting. The design of the attack path or fingerprint of APTs using threat modeling approaches has as its goal as the exploration of attacks on a system and discovering the system vulnerabilities. It helps security analysts and system specialists to analyze the design from the attackers’ perspective in order to better understand APT’s TTP [191]. Fingerprinting is a collection of information about a cyber-threat that identifies the Tactic, Technique, and Procedure (TTP) utilized to perpetrate the attack [8]. These fingerprints can be handled from different sources such as mobile device resource usage (such as CPU, memory, etc.) and user activity [12]. A generalized attack path or fingerprint is required to simplify the TTP of the APT. For example, different mobile APT malware such as Android/Chuli.A and Riltok [183] have initiated using spear-phishing attacks. A generalized fingerprint is required for these malwares as they have different TTPs in order to simplify the training process for the learning model.

#### 5.2.4. To Manipulate Mobile Device Behavior through Resource Usage and User Activity

There is a need for a risk and trust management model that identifies assets and threats to these assets and quantifies the likelihood and impact of the APT. This proposed model first continuously monitors and quantifies the device’s behavioral sources (such as CPU, memory, etc.) and user activity [12], then compares the quantified results with the generalized attack paths in order to detect mobile APT and prevent the increasingly severe risk of data leakage [21] and lateral movement [22].

One example is when an APT attacker attempts to compromise the targeted system, the zero trust model is utilized to authenticate the only legitimate user to access the asset. If the APT attacker successfully infiltrates the device and tries to obtain user credentials by targeting file systems and registries, this is reflected in the CPU utilization and triggers the risk. By using the risk assessment approach, mobile APTs can be detected by quantifying the CPU utilization and comparing it with the generalized attack paths, and responding to the APT activity.

#### 5.2.5. To Design an APT Solution That Is personalized Based on Mobile Users

The purpose of this solution is to determine the risk faced by each mobile user as the risk of each device’s behavior varies according to the user’s behavior [18]. User behavior may be described as the actions of a mobile user, whether malicious or not, that contribute to APT attacks [8]. One example is users A and B using the same mobile application. Although they both utilize the same application, each user faces different security risks. This is because of how the user is using the application, not how the application works [18].

## 6. Proposed Conceptual APT Mitigation Framework

As discussed in Section 5.1, most of the APT defense solutions have failed to tackle an APT issue. In this section, the authors propose a conceptual framework of a mobile device behavior fingerprint for APT mitigation. This framework is a novel and most promising [12] in the fight against APT, which helps the security analysts to make a precise decision in detecting any suspicious incidents related to APT. It is a multilayered/multiphase comprehensive APT detection and protection framework that follows the NIST cyber security framework. Within this framework, Cyber-Cognitive Situation Awareness (CCSA) is used. CCSA is self-awareness that is capable of acquiring the following three properties at execution time: (1) Auto-reflective: It is aware of its software architecture, hardware infrastructure, and execution environment in order to meet its operational goals, (2) Auto-predictive: It is capable of predicting the effects of a dynamic change caused by potential adaptive actions, and (3) Self-adaptive: It has the ability to meet its operating goals despite changes in the environment.

OODA loop is a CCSA model that has the ability to monitor and capture different types of threats, analyze them, and devise a plan to mitigate further threats [190]. Its purpose is to resolve the APT issue and increase awareness about the surrounding environment.

**Figure 12:**
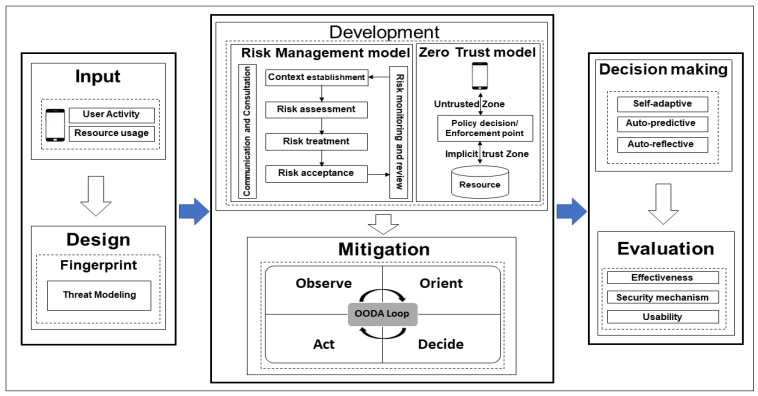
Conceptual framework of mobile device behavior fingerprint for APT mitigation.

As shown in Figure 12, the OODA loop has four phases, Observe–Orient–Decide–Act [190]. Each phase in an OODA loop is a process that interacts with its environment.

### 6.1. Observe

In this phase, after collecting the behavioral source data, such as the external and in-device behavior sources for each smartphone user, such as resource usage (CPU, memory, battery, and network), design the generalized attack paths or fingerprints using threat modeling approaches from the collected behavioral source data. These generalized attack paths are used to train the risk and trust assessment model in order to detect unknown mobile APTs during the testing process. For example, different mobile APT malware such as Android/Chuli.A and Riltok [183] have initiated using spear-phishing attacks. A generalized fingerprint is required for these malwares as they have different TTP in order to simplify the training process for the learning model.

### 6.2. Orient

In this phase, the risk and trust assessment model is used. The risk assessment model is used to continually monitor and quantify the behavioral source data such as the CPU, memory, battery, and network. These quantified behavioral data are compared with the generalized attack paths in the training process to detect and respond to any suspicious activity. While the zero trust model is used to allow only authorized users to access their resources regardless of their location, this model is used to prevent the increasingly severe risk of data leakage [21] and lateral movement [22].

One example is when an APT attacker attempts to compromise the targeted system, where the zero trust model is utilized to authenticate the only legitimate users to access the asset. If the APT attacker successfully infiltrates the device and tries to obtain user credentials by targeting file systems and registries. This is reflected in the CPU utilization and triggers the risk. By using the risk assessment approach, mobile APT can be detected by quantifying the CPU utilization and comparing it with the generalized attack paths and responding to the APT activity.

### 6.3. Decide

The most justified and appropriate measure for the current situation is chosen for implementation to achieve the Confidentiality, Integrity, and Availability (CIA) of the asset.

### 6.4. Act

Implementing the action in the decide phases such as preventing the APT lateral movement and data leakage. After the Act phase, the loop continues back to the Observation phase to observe and detect the APT on the device’s behavior. Finally, the APT mitigation framework will be evaluated regarding effectiveness, security mechanisms, and usability.

## 7. Study Limitations

This review has several limitations. First, this study is constrained by the search keywords and the publication date (2011–2022). Second, we used a small number of electronic sources such as SCOPUS, Science Direct, IEEE Xplore, ACM, and Springer. In addition, our research included only English language journal articles, and we cannot ensure that we included all relevant studies in our review.

## 8. Conclusions

This study delved into the cybersecurity APT defense solutions using different mechanisms such as situational awareness, risk management, trust management, and artificial intelligence by implementing a systematic literature review. Due to the rapid growth of mobile devices in a variety of fields, massive volumes of data are constantly generated, necessitating a greater emphasis on privacy and security. APT features can be viewed through stages using threat modeling frameworks such as MITRE. If these attacks succeed, the attacker could manipulate the device’s behavior, applications, and services based on its goal, be it data theft or sabotage. Such manipulations lead to signifying a deviation from a known behavioral baseline that can then be utilized for the detection of suspicious incidents. With the rapid expansion of cyber threats such as APT, conventional methods for improving mobile security have become outmoded. An alternative solution is device behavior fingerprinting, which can be considered one of the most promising approaches to mitigate mobile APT.

The authors summarized, categorized, and mapped the existing literature on APT features, APT defense mechanisms, and risk management models using formulated research questions. For the survey, 112 papers from (2011 to 2022) were carefully selected and evaluated using the PRISMA approach. In addition, the authors proposed a conceptual framework of mobile device behavior fingerprinting for APT mitigation. This framework is auto-reflective, auto-predictive, and self-adaptive. Finally, the SLR validates device behavior fingerprinting as a potential technique for ensuring security and privacy in mobile environments.

## Figures and Tables

**Table 1:** Differences between APTs and traditional malware attacks.

Characteristics	Advanced Persistent Threats	Traditional Malware Attacks
Attack definition	APT is a highly sophisticated, well-organized, and well-targeted attack (e.g., Stuxnet).	The term “malware” refers to software intended to attack and disrupt digital systems (e.g., ransomware).
Attacker	Government actors and organized criminal groups	A cracker (a hacker in illegal activities).
Target	Targets a wide range of businesses and organizations, including diplomatic organizations, the information technology sector, and others.	Targets any personal or business device.
Purpose	The purpose of this attack is to damage a specified target or steal sensitive data.	The purpose of this attack is financial gain.
Attack life cycle	Maintain persistence as possible using different conceal tools.	The malware is eliminated when it is identified via security tools (e.g., anti-virus software).

**Table 2:** Situational awareness models developed to provide quantitative indicators in decision-making.

Model	Focus
SAM (Situational Awareness Model)	Cognitive decision-making
OODA Loop (Observe–Orient–Decide–Act)	Cognitive decision-making
JDL DFM (JDL Data Fusion Model)	Processing and fusion of data and SA
CSAM (Cyber Situational Awareness Model)	Business continuity planning and CSA
SARM (Situational Awareness Reference Model)	Situational awareness
ECSA (Effective Cyber Situational Awareness)	CSA in computer networks

**Table 3:** PICOC criteria.

Population	APT Attack Defense
Intervention	APT defense mechanisms
Comparison	Not available
Outcomes	Device behavior-based APT detection
Context	Review the existing studies of device behavior-based APT detection

**Table 4:** Mapping between the collected APT features and the ATT&CK-based taxonomy: from Initial Access to Impact stage.

References	APT Features	ATT&CK
[1,3,7,28,79,80,81,82,83,84,85,86,87,88,89,90,91,92,93,94,95,96,97,98,99,100]	Spear phishing	Initial access
[3,28,79,84,88,99,101,102]	Watering hole	
[3,28,79,84,88,99,101,102]	Malware	
[1,3,88,89,102,103,104,105]	Application repackaging	
[106]	Attacks on an Internet-facing server	
[3,83,89,101]	Removable device	
[3,89,107]	Drive-by download	
[96]	Spoofing attack	
[7,82,108]	SQL injection	Execution
[3,5,82,84,86,87,88,90,94,97,101,109,110,111,112,113,114]	Zero day, known vulnerability	
[79,101,115]	Remote code execution/Code injection	
[116]	User to Root (U2R)	Persistence
[116]	User to Root (U2R)	Privilege escalation
[6]	Unauthorized access	Defense evasion
[108]	Buffer overflow	
[28]	Brute force	Credential access
[28]	Pass hash	
[79,82,83,117,118]	Man-in-the-middle	
[119]	Password cracking	
[120]	Eavesdropping	
[78,80,81,82,85,87,97,105,107,111,121,122,123]	Social engineering	Discovery
[124]	Probe	
[100,125]	Lateral/Internal spear-phishing emails	Lateral movement
[108]	Data leakage	Collection
	Cloud data leakage.	
[126]	Removable device	C&C and Exfiltration
	Tunneling over protocol	
[3,76,79,81,92,97,111,115,124,125,126,127,128,129,130]	DOS	Impact
[4,82,131]	Botnet	
[108]	Software update	
	Data fabrication	

**Table 5:** APT defense mechanisms.

Technique Used	Component	Platform	APT Defense Mechanisms
Global abnormal forest (GAF) [3]	Network	Mobile and computer	D
Mobile secure manager (MSM), analyzer (static and dynamic analysis) [132]	Human behavior	Mobile	D
Federated learning algorithm [5]	Application	Mobile	D
Naïve Bayes classifier [28]	Application	IoT	D
Domain generation algorithm (DGA) [79]	Network	IoT	D
Deep autoencoder [6]	Network	IoT	D
Genetic programming, classification and regression trees, support vector machines, and dynamic Bayesian game model [1]	Network	IoT	D
Maximum connected subgraph algorithm [7]	Network	IoT	D
AutoEncoder and 1D CNN (1-Dimension Convolutional Neural Network) [81]	Application	IoT	D
Prospect Theoretic Game [82]	Network	IoT	D
Random forest (RF) [83]	Network	Unmanned aerial vehicles (UAVs)	D
Outlier Dirichlet Mixture (ODM-ADS) mechanism [133]	Network	Fog computing	D
Random forest (RF), support vector machine (SVM), and multi-layer perceptron (MLP) [134]	Network	General	D
Multi-layer perceptron (MLP), convolutional neural network (CNN), and long short-term memory (LSTM) [103]	Network	General	D
Cumulative prospect theory (CPT) [135]	Network	General	D
Malicious IP address detection module (MIPD), malicious Secure Sockets Layer (SSL) certificate detection module (MSSLD), domain-flux detection module (DFD), and Tor connection detection module (TorD) [84]	Network	General	D
Semantic event correlation [117]	Device and Network	Computer	D
Dynamic programming algorithm [105]	Device	Computer	D
Support vector machine (SVM) [136]	Network	Computer	D
Signature-based and anomaly-based detection technology [131]	Network	Computer	D
Threat detection (disguised executable file detection (DeFD), malicious file hash detection (MFHD), malicious domain name detection (MDND), malicious IP address detection (MIPD), malicious SSL certificate detection (MSSLD), domain flux detection (DFD), scan detection (SD), and Tor connection detection (TorCD)) Alert correlation (Alerts filter (AF), clustering of alerts (AC), and correlation indexing (CI)) Attack prediction (machine-learning-based prediction module (PM)) [137]	Network	Computer	D
Decision tree [138]	Device	Computer	D
Memory-augmented deep auto-encoder (MemAE) [130]	Network	Computer	D
Random forest classifier [85]	Application	Computer	D
Vermiform window, scalable inference engine called SANSA, and ontology-based data abstraction [109]	Device	Computer	D
Bayesian networks [139]	Network	General	D
Random forest algorithms [110]	Application	IoT	D
Random forest classifier [86]	Application	Computer	D
Self-organizing feature maps [124]	Application	Computer	D
Vectorized mobile ATT&CK matrix and the indicator pairing technique [87]	Application	Mobile	D
Random forest (RF) [140]	Network	IoT	D
Manhattan distance and metric distance algorithms [88]	Application	Computer	D
Random forest and isolation forest [101]	Application	Computer	D
Passive network monitoring, in-host auditing subsystem monitoring [89]	Network and device	General	D
Federated learning algorithm, differentially private data perturbation mechanism [141]	Network	IoT	D
Hierarchical clustering algorithm [90]	Network	IoT	D
Reconnaissance deception system (RDS) [142]	Network	Computer	M
Hidden Markov model (HMM) [143]	Network	IoT	M
Pretense theory [144]	Network	Cloud computing	M
Metagames and hypergames [145]	Network	Computer	M
Data-centric security approach–Ciphertext Policy-Attribute-based Encryption(CP-ABER-LWE) scheme [4]	Device	IoT	P
Analytic hierarchy process (AHP) and the Technique for Order Preference by Similarity to Ideal Solution (TOPSIS) model, and the OpenFlow technique [125]	Network	General	P
Lyapunov-based intelligence-driven security-aware defense mechanism [121]	Network	Computer	P
Trusted Platform Module [118]	Network	Computer	P
Cyber risk management (cyber-insurance) and game theory (dynamic Stackelberg game) [111]	Network	Fog computing	I
Cyber risk management (cyber-insurance) and game theory (FlipIn game) [107]	Network	IoT	I
Role- and attribute-based access control and multilevel security model [102]	Device	Mobile	P
J48, Boyer-Moore algorithm, and k-NN (k Nearest Neighbor) algorithm [116]	Network	Computer	D&R
Attack-defense trees (ADT) approach [146]	Network	Computer	I
Bayesian network model [91]	Network	Cloud computing	P
Strategic trust, game theory (signaling game and the FlipIt game) [92]	Network	Computer	P
Multi-layer framework (iSTRICT) and associated equilibrium concept (GNE), and an adaptive algorithm [112]	Network	IoT	P
Security information event management system (IBM Q-radar) [93]	Network	General	I
Individual-level continuous-time dynamic model [147]	Network	Computer	D
Zero-day attacks activity recognition method, malicious C&C DNS mining method (MCCDRM), and purpose-oriented situation-aware access control [113]	Network	IoT	D
Adaboost classifier [148]	Network	IIoT	D
AutoEncoder [149]	Network	Computer	D
Bayesian classification algorithm and fuzzy analytical hierarchy process [150]	Network	General	D
Bayesian Stackelberg game [151]	Network	General	D
Hypergame theory [152]	Network	General	M

APT Defense mechanisms: D = Detection, P = protection, I = Identification, R = Response, M = Mitigation.

**Table 6:** Risk management approaches.

Approach	Platform	Attack Type
Opportunity-enabled risk management (OPPRIM) methodology [153]	Mobile	Cyber-attack
Permission-based Hybrid Risk Management framework for Android apps (PHRiMA) [154]	Mobile	privilege-induced attack
Bi-level game-theoretic framework [107]	IoT	APT
Intelligent risk management framework [146]	IoT	DDOS and SQL injections attacks
IoT security risk management strategy reference model (IoTSRM2) [155]	IoT	Cyber-attack
IoT risk management model [156]	IoT	Cyber-attack
IoT security risk model [157]	IoT	Cyber-attack
Threat and risk management framework [158]	IoT	Cyber-attack
Proactive CAV cyber-risk classification model [159]	Connected and Autonomous Vehicle (CAV)	Cyber-attack
Cyber risk management (cyber-insurance) tool [160]	Fog computing	APT
Cyber risk vulnerability management (CYRVM) platform [161]	General	Cyber-attack
Bi-level mechanism [162]	General	Cyber-attack
AMBIENT (Automated Cyber and Privacy Risk Management Toolkit) [163]	General	Cyber-attack
Information security risk management situation aware ISRM (SA-ISRM) model [61]	General	Cyber-attack
Risk and dynamic access control tool [164]	General	Cyber-attack
Knowledge security risk management model [165]	General	Cyber-attack
Information security risk management (ISRM) [166]	General	Cyber-attack
Semi-Markov decision process framework [167]	5G edge-cloud ecosystem	(DoS) attack
Risk management framework [168]	Cyber-physical systems	Cyber-attack
Integrated cyber-security risk management framework [169]	Cyber-physical Systems	Cyber-attack
Security information event management system (IBM Q-radar) [93]	General	APT
Cyber risk management (cyber-insurance) and dynamic Stackelberg game [111]	Fog computing	APT
Viewnext-UEx model [170]	Computer	Cyber-attack
